# Antifungal Activity, Synergism with Fluconazole or Amphotericin B and Potential Mechanism of Direct Current against *Candida albicans* Biofilms and Persisters

**DOI:** 10.3390/antibiotics13060521

**Published:** 2024-06-03

**Authors:** Peihui Zou, Jia Liu, Peng Li, Qingxian Luan

**Affiliations:** Department of Periodontology, Peking University School and Hospital of Stomatology & National Center for Stomatology & National Clinical Research Center for Oral Diseases & National Engineering Research Center of Oral Biomaterials and Digital Medical Devices & Beijing Key Laboratory of Digital Stomatology & NHC Key Laboratory of Digital Stomatology & NMPA Key Laboratory for Dental Materials, Beijing 100081, China; zphntyc@163.com (P.Z.); dentistliujia@126.com (J.L.)

**Keywords:** direct current, *Candida albicans*, persisters, reactive oxygen species, superoxide dismutase

## Abstract

*Candida albicans*, as a notorious fungal pathogen, is associated with high morbidity and mortality worldwide due to its ability to form biofilms and persisters that can withstand currently available antifungals. Direct current (DC) has demonstrated a promising antimicrobial effect and synergistic effect with antimicrobials against various infections. Here, we first found DC exerted a killing effect on *C. albicans* planktonic and biofilm cells. Moreover, DC showed a synergistic effect with fluconazole (FLC) and amphotericin B (AMB). Notably, near-to-complete eradication of AMB-tolerant *C. albicans* biofilm persisters was achieved upon DC treatment. Next, the mechanism of action of DC was explored through mapping the genes and proteomic profiles of DC-treated *C. albicans*. The multi-omics analysis, quantitative real-time PCR and assay of reactive oxygen species (ROS) demonstrated DC exerted an antifungal effect on *C. albicans* by increasing cellular oxidative stress. As revealed by multiple analyses (e.g., protein assay based on absorbance at 280 nm and rhodamine 6G assay), DC was able to enhance membrane permeability, inhibit drug efflux and increase cellular FLC/AMB concentration of *C. albicans*, thereby mediating its synergism with the antifungals. Furthermore, DC inhibited superoxide dismutase 2 (SOD2) expression and manganese-containing SOD (Mn SOD) activity, leading to ROS production and enhanced killing of *C. albicans* biofilm persisters. The current findings demonstrate that the adjunctive use of DC in combination with antifungals is a promising strategy for effective control of *C. albicans* infections and management of antifungal resistance/tolerance in *Candida* biofilms.

## 1. Introduction

*Candida albicans* is a predominant commensal and primary pathogenic fungus responsible for various mucosal and systemic infections, particularly in immunocompromised individuals. These infections have become a significant public health concern due to their increasing prevalence, high morbidity and mortality rate [1,2]. The fungus is often prone to formation of biofilms with increased antifungal resistance on indwelling medical devices and mucosal surfaces [3,4]. Moreover, as previously described by us and other researchers, *C. albicans* biofilms can form a subpopulation of antifungal-tolerant persisters that remain viable and cannot be completely eradicated by antifungals even at lethal concentrations [3,5,6]. Notably, the persisters existing in the biofilms are capable of reviving and repopulating the biofilms once antifungal treatment stops. It is believed that the ability of *C. albicans* to form biofilms and persisters is the underlying reason for sustained and recurrent infections and therapeutic failure [1,7]. It has been demonstrated that the currently available antifungals such as the azole antifungal fluconazole (FLC) and the polyene antifungal amphotericin B (AMB) cannot readily control the biofilms and their persisters [8,9]. Therefore, a novel effective antifungal strategy is urgently needed to tackle this challenge.

Electric current has recently drawn much attention for its ability to exert a direct killing effect and enhance the action of antimicrobials against biofilms, frequently in the form of low amplitude (1000 μA or less) direct current (DC) [10,11,12]. DC is a form of electric current that flows only in the same direction [13]. Our previous studies have confirmed the synergistic effect of DC with antibiotics in killing a keystone periodontal pathogen *Porphyromonas gingivalis* and subgingival plaque biofilms in vitro [14,15]. The disruption of membrane integrity as well as the generation of chlorine, hypochlorous acid, oxygen and reactive oxygen species (ROS) may play an important role in the DC-induced killing effect [14,16]. Currently, neither the antifungal effect of DC nor its synergistic effect with antifungals on *C. albicans* biofilms/persisters are well-studied, and the underlying mechanisms are largely unknown [17]. The application of DC provides a promising approach to control biofilms that display increased resistance [12,18]. Hence, the aim of this study was to investigate the DC-killing effect and coordinated action of DC with FLC and AMB on *C. albicans* biofilms and their persisters, as well as the underlying mechanisms. 

## 2. Materials and Methods

### 2.1. Candida Strain, Culture, Compounds and Stimulator

A well-characterized clinical isolate used in our previous studies, *C. albicans* BF-1, was selected for this study [5,19]. For broth culture, the yeast was incubated on yeast extract peptone dextrose (YPD) media (Solario, Beijing, China). For solid culture, it was grown on Sabourauds dextrose agar (SDA, Hopebio, Qingdao, China) plate for 24 h. Fungal growth was monitored by measuring the optical density (OD_600_) in SpectraMax Multimode Microplate Reader (Molecular Devices Ltd., San Jose, CA, USA). The suspension was adjusted to OD_600_ of about 0.08 (1 × 10^7^ colony-forming units (CFU)/mL) and placed on 24-well plates (Corning, Corning, NY, USA) for 72 h to form biofilms. The biofilms in the bottom of each well were harvested by sonication (100 W) for 1 min and mechanical scrape with sterile pointed probe, and suspended in liquid medium. 

Stock solutions of fluconazole (FLC) (Sigma-Aldrich, St. Louis, MI, USA) and polyene amphotericin B (AMB) (Sigma-Aldrich, USA) were dissolved in dimethyl sulfoxide (DMSO) (Solario, China). 

The DC-stimulator apparatus (KuShi Medical Technology Co.Ltd., Shanghai, China) was described in our previous study [14]. The whole circuit maintained constant current intensity of 1000 μA by means of a DC output chip, with one 5-voltage lithium battery and two carbon electrodes inserted into the fungal solution. 

### 2.2. Experiment Design

#### 2.2.1. The Killing Effect of FLC/AMB on *C. albicans* in Planktonic State and Biofilm State

Various concentrations (0.25–1280 μg/mL) of FLC/AMB were used to stimulate planktonic *C. albicans* (10^7^ CFU/mL, for 12 h) and the 72 hour-old *C. albicans* biofilms (for 24 h). The viable fungi were calculated by CFU counting and observed by LIVE/DEAD staining. 

#### 2.2.2. The Killing Effect of 1000 μA DC on *C. albicans* in Planktonic State and Biofilm State

Planktonic *C. albicans* (2 mL 10^7^ CFU/mL) was stimulated by 1000 μA DC for 1, 6 and 12 h. *C. albicans* biofilms were stimulated by 0, 1000 μA DC for 24, 48 and 72 h. The treated *C. albicans* was analyzed by viable fungi count, cell metabolic activity assay, scanning electron microscopy (SEM) and LIVE/DEAD staining.

For examining the role of ROS in the DC-induced antifungal effect, *C. albicans* was stimulated by 1000 μA DC, Tempol (Solario, China) alone or 1000 μA DC combined with Tempol (Solario, China) for 12 h. The concentrations of Tempol used for the planktonic state and biofilm state were 15 μM and 30 μM, respectively. Afterwards, the treated *C. albicans* was analyzed by ROS assay, CFU counting, gene expression assay, total SOD assay and fluorescence microscopy.

#### 2.2.3. Synergistic Effect of DC with FLC/AMB on *C. albicans* in Planktonic State and Biofilm State

The *C. albicans* was stimulated by 1000 μA DC, FLC and AMB separately or 1000 μA DC combined with FLC or AMB for 6 h (in planktonic state) or 12 h (in biofilm state). The concentration of FLC/AMB for the planktonic state and biofilm state were 1 μg/mL and 8 μg/mL, respectively. Subsequently, the treated *C. albicans* was analyzed by CFU counting and LIVE/DEAD staining.

#### 2.2.4. The Impact of DC on Membrane Permeability and Intracellular Drug Concentrations of *C. albicans*

The planktonic *C. albicans* was stimulated by 1000 μA DC for 0.5, 1, 6 and 12 h. Afterwards, the treated *C. albicans* was analyzed by A280 assay, electrical conductivity assay, transmission electron microscopy (TEM), HPLC-DAD/MS and gene expression assay. The rhodamine 6G (Rh6G) absorption and efflux assay was used to determine the transport activity after exposure to DC for different time periods.

#### 2.2.5. The Role of SOD2-Mn SOD Pathway in the Killing Effect of DC on AMB-Tolerant Persisters of *C. albicans* Biofilms

The *C. albicans* biofilms were stimulated by 256 μg/mL AMB, MnTMPyP (10 μM) and 1000 μA DC separately or combined with MnTMPyP, or combined with both MnTMPyP and AMB for 24 h. Afterwards, the treated biofilms were subjected to the persister assay, gene expression assay, total ROS assay, mitochondrial ROS (mtROS) assay and Mn SOD activity assay.

### 2.3. Antimicrobial Susceptibility and MIC Testing

The minimum inhibitory concentration (MIC) of FLC and AMB against *C. albicans* was determined by broth microdilution method following M27-A3 guidelines of the Clinical and Laboratory Standards Institute [20]. Serial 2-fold dilutions of FLC and AMB (100 μL, ranging from 0 to 128 μg/mL) were made in culture media in 96-well plates (Corning, USA). The MIC was the lowest concentration that showed no visible growth. The experiment was repeated three times.

### 2.4. Persister Assay

The persister assay was performed as described previously [5,21]. Briefly, 72 hour-old *C. albicans* biofilms were exposed to 256 μg/mL AMB for 24 h at 37 °C. The biofilm cells were washed two times with PBS and harvested. After serial dilution, the biofilm cells were spread onto SDA plates for further counting. To confirm that the surviving colonies were persister cells, two colonies were reinoculated into fresh YPD medium and an in vitro susceptibility assay was performed to ensure that the MIC had not changed. The survival rate of persisters was calculated on the basis of viable cells out of total cells in an untreated culture.

### 2.5. Viable Fungi Counting

Viable fungi counting in planktonic or biofilm states was calculated by colony forming units (CFU) assay. After the treated *C. albicans* was transferred to liquid medium and serially diluted, 100 μL suspension was plated on blood agar by L-shape sterile coating bar (BIOLOGIX, Jinan, China) for further counting.

### 2.6. Cell Metabolic Activity Assay and Crystal Violet Staining 

The cell metabolic activity of *C. albicans* biofilms was measured using an enhanced Cell Counting Kit-8 (CCK-8, Beyotime, Shanghai, China), as described by the manufacturers [22]. A volume of 100 μL of stained solution was measured in 96-well plates (Corning, USA) at 450 nm. The crystal violet staining was performed as previously described [14].

### 2.7. LIVE/DEAD Staining and Fluorescent Microscopy Observation

The viability of *C. albicans* was further evaluated by live/dead staining and fluorescent microscopy observation. After being stained with mixed CFSE (1 μM, live cells indicator stained green, AbMole, China) and PI solution (1 μM, dead cells indicator stained red, AbMole, China) for 20 min in the dark, the stained *C. albicans* was observed by fluorescent microscopy (Leica Microsystems, Germany), and images were obtained.

### 2.8. Detection of Intracellular ROS, mtROS, Total SOD Activity and Mn SOD Activity

2′,7′-Dichlorodihydrofluorescein diacetate (H2DCF-DA) (Solario, China) and MitoSOX Red (Invitrogen, Waltham, MA, USA) were used to detect total intracellular ROS and mtROS, respectively, as previously described. The ROS content in *C. albicans* was determined by fluorescence intensity. The treated *C. albicans* cells were washed and stained with DCFH-DA (10 μM) or MitoSOX Red (5 μM) for 20 min in the dark. After that, the cells were centrifuged (3000× *g*, 5 min) and washed two times with PBS. The relative fluorescence intensity (RFU) of 100 μL stained cells was detected by an Enspire microplate reader (PerkinElmer, Rodgau, Germany) at an excitation of 488 nm and an emission of 530 nm. 

The total SOD activity and Mn SOD activity were evaluated by SOD assay kit and Mn-SOD Assay Kit with WST-8 (Beyotime Institute of Biotechnology, Shanghai, China) according to the manufacturer’s instructions, respectively [23]. The absorbance was assessed at 450 nm using a microplate reader. 

### 2.9. SEM Observation and TEM Observation

The samples of *C. albicans* biofilms formed on silicon wafers (Zhejiang Lijing Silicon Material Co., Quzhou, China) were washed with sterile PBS, and fixed with 2.5% (*v*/*v*) glutaraldehyde (Solario, China) for 2 h. Subsequently, the biofilms were dehydrated for 10 min using an ethanol gradient (50%, 60%, 70%, 90% and 100%). Finally, the samples were coated with gold and observed under SEM (Hitachi, Japan).

The detailed sample preparation before TEM observation was the same as in a previous study [24]. After fixation, dehydration with 30%, 50%, 70%, 90% and 100% ethanol, embedding and polymerization, the ultrathin sections (65 nm) were cut and stained. All sections were examined with transmission electron microscope JEM 1400 (JEOL Co, Tokyo, Japan).

### 2.10. RNA-Seq, Quantitative Proteomics Using TMT Labelling and Bioinformatics Analysis

All treated cells were collected and centrifuged at 3000× *g* for 10 min. The total RNA and protein samples were sent to Allwegene Technology Company Limited (Beijing, China) for further processing and bioinformatics analysis. The detailed process was performed as previously described [25,26]. For RNA-Seq, *p* value < 0.005 and |log2(fold change)| ≥ 1 were set as the threshold for significantly differential expression. For proteomics, TMT coupled with liquid chromatography electrospray ionization tandem mass spectrometry (LC-MS/MS) analysis was referenced to the database of uniprot_*Candida_albicans*_strain_SC5314 _6041_20230218.fasta. The expression fold change was > 1.2-fold (up-regulation greater than 1.2-fold or down-regulation less than 0.83-fold) and *p* value was < 0.05 (*t*-test or others).

### 2.11. Quantitative Real-Time PCR (qPCR)

1 μg of total RNA was extracted using the Fungal Total RNA Isolation Kit (Sangon Biotech, China). cDNA was synthesized with a Reverse Transcription System (Toyobo, Osaka, Japan) and detected using the Starlighter SYBR Green qPCR Mix (Foreverstar Biotech, Beijing, China) in a 7500 Real-Time PCR System (Applied Biosystem, San Francisco, CA, USA). The q-PCR was carried out for 35 cycles of 94 °C for 1 min, 60 °C for 1 min, and 72 °C for 1 min. Relative gene expression was calculated by the CT method using ACT1 as the reference gene. Experiments were performed in biological duplicates and measured in technical triplicates. Primers used for real-time PCR are given in Table S1 in the Supplementary Materials.

### 2.12. A280 Absorbance Assay and Electrical Conductivity Assay

Protein concentration was determined via the A280 absorbance assay. After exposure to DC for 0.5, 1, 6 and 12 h, 2 μL supernatant of the treated *C. albicans* was taken to measure the absorbance at 280 nm using a NanoDrop instrument (Thermo Scientific NanoDrop, Wilmington, DE, USA).

The change of electrical conductivity is an important index to measure the change of fungi cell structure. The supernatant of the treated *C. albicans* was taken to measure its conductivity using the DDS–307 conductivity meter (Shanghai instrument, Shanghai, China).

### 2.13. Rh6G Absorption and Efflux Assay

In this study, we used the Rh6G tracer method to explore whether DC interfered with the drug absorption and efflux activity [27]. Planktonic *C. albicans* was grown to the exponential phase. After centrifugation (3000× *g*) for 10 min, the yeast cells were harvested and washed twice with glucose-free PBS (Solario, China) and resuspended to 1 × 10^7^ CFU/mL. After oscillated in the shaker for 1 h to deplete cellular energy, 10 μM Rh6 G was added in the *C. albicans* suspension for the subsequent experiment. The *C. albicans* cells were resuspended in 1 mL medium and 100 μL diluted suspensions were measured at timepoint of 0, 10, 20, 30 and 50 min by microplate reader. For the Rh6G efflux, the treated *C. albicans* was placed in an ice bath for 10 min; then, after centrifugation (3000× *g*) for 10 min, the yeast cells were harvested and washed twice with glucose-free PBS. Subsequently, the intracellular Rh6 G was measured every 30 min. The Rh6 G-alone group was set as the control group. Then, the fluorescence intensity of intracellular Rh6G was measured with excitation at 488 nm and emission at 530 nm.

### 2.14. High-Performance Liquid Chromatography Mass Spectrometry (HPLC-DAD/MS) Analysis for Intracellular FLC and AMB

*C. albicans* cells were centrifuged at 3000× *g* for 5 min at 4 °C and washed with cold PBS twice. Similarly to Section 2.13, the supernatant was removed and centrifugation was repeated once. The sample processing was performed as previously described [24]. FLC and AMB were detected using authentic samples and mass ion peaks at 307.110 [M + H]^+^ and 924.079 [M–H]^−^.

### 2.15. Statistical Analysis

The data were presented as mean ± standard deviation (SD), and all experiments were performed three to six times. Significant differences between two or multiple groups were analyzed by the two-tailed Student’s *t*-test or one-way ANOVA, in which post-hoc analysis was performed with the Tukey test. Data were analyzed by GraphPad Prism 9.4.1 software (GraphPad Software Inc., La Jolla, CA, USA). Differences were considered significant at a *p* value of < 0.05.

## 3. Results

### 3.1. The Dose-Dependent Effect of FLC/AMB against Planktonic and Biofilm States of C. albicans

**Figure 1:**
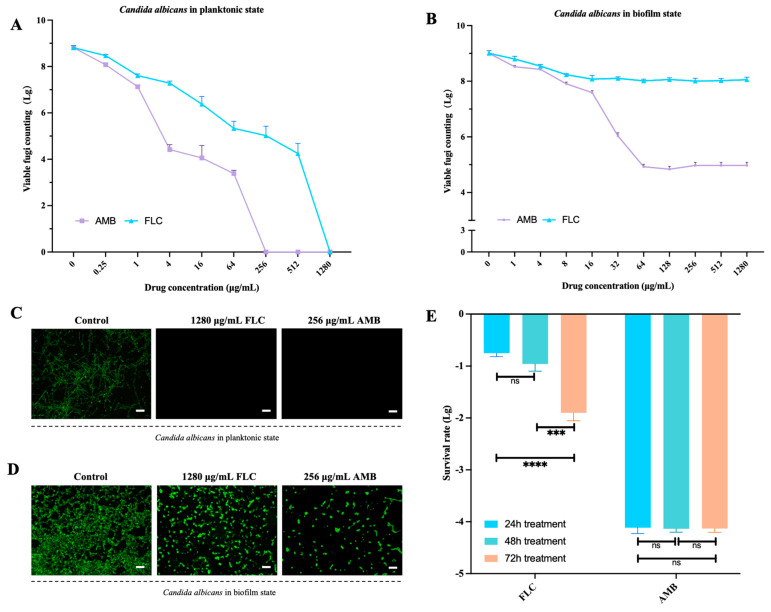
Dose-dependent inhibition of *C. albicans* BF-1 in planktonic state and biofilm state by FLC and AMB. Planktonic *C. albicans* were treated with various concentrations of FLC/AMB for 12 h. Next, the colonies were counted after 1 day of incubation (**A**). *C. albicans* biofilms were treated with various concentrations of FLC/AMB for 24 h. Next, the colonies were counted after 1 day of incubation (**B**) and recultured for the same stimulation and assay twice. The surviving cells were assessed using live/dead staining (scale bar 50 μm, (**C**) in planktonic state and (**D**) in biofilm state). The survival rate of *C. albicans* biofilms was calculated for comparison (**E**). *** and **** denote significant difference of *p* < 0.001, *p* < 0.0001, respectively. ns denotes no significant difference.

The MIC of FLC and AMB against planktonic *C. albicans* were 4 μg/mL and 0.5 μg/mL, and the minimum lethal concentration reached 1280 μg/mL and 256 μg/mL, respectively (Figure 1A). As for biofilms, the amount of viable *C. albicans* in biofilms decreased by less than 1-log killing as FLC concentration increased up to 16 μg/mL, and remained at a similar level even at higher concentrations (32–1280 μg/mL). As the concentration of AMB increased, the treatment killed the majority of *C. albicans* biofilms but failed to eradicate a tiny subpopulation of the biofilms, presenting a biphasic pattern. The fractions of biofilm persisters reached 0.01% even at lethal doses of AMB (64 to 256 μg/mL, Figure 1B). After consecutive treatment of the biofilms by FLC (1280 μg/mL) or AMB (256 μg/mL) for another 72 h, the survival rate of AMB-treated biofilms was maintained at a relatively stable level and the MIC value did not show obvious changes, while the survival rate of FLC-treated biofilms decreased significantly as exposure times increased to 72 h and the MIC value did not change (Figure 1C–E).

### 3.2. The Time-Dependent Killing Effect of 1000 μA DC against Planktonic and Biofilm States of C. albicans

**Figure 2:**
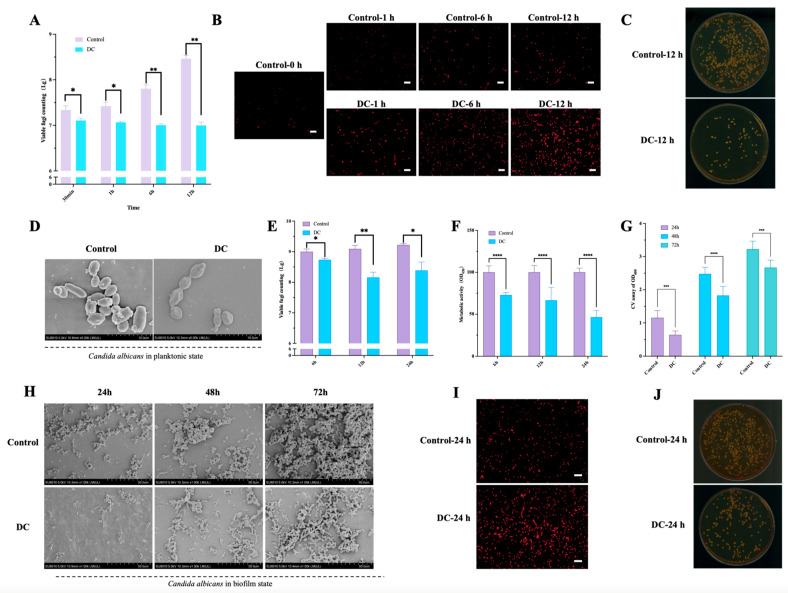
The killing effect of 1000 μA direct current (DC) with different stimulus durations on *C. albicans* in planktonic state and biofilm state. Viable fungi counting (**A**) and dead staining (stained red, (**B**)) after exposure to DC for 1 h, 6 h and 12 h in planktonic state. The SDA agar plate with *C. albicans* colonies (**C**) and scanning electron microscopy (SEM) observation (**D**) after exposure to DC for 12 h in planktonic state. Viable fungi counting (**E**) and metabolic activities assay (**F**) after exposure to DC for 6, 12 and 24 h. Total biofilm amount (**G**) and SEM observation at magnifications of ×1000 (scale bar 50 μm,(**H**)) at 24, 48 and 72 h of DC treatment. SEM observation at magnifications of ×5000 (scale bar 10 μm, (**I**)) , dead staining (stained red, scale bar 50 μm, (**F**)) and SDA agar plate with *C. albicans* colonies (**J**) after exposure to DC for 24 h. *, **, ***, **** denote significant difference of *p* < 0.05, *p* < 0.01, *p* < 0.001, *p* < 0.0001, respectively.

For the planktonic *C. albicans*, compared to untreated control, the CFU counting was significantly decreased when treated with 1000 μA DC for 0.5 h, 1 h, 6 h and 12 h (Figure 2A), which reached 0.23, 0.35, 0.80 and 2.00-log killing, respectively. The dead staining showed consistent viable fungi count results (Figure 2B). The agar plate of *C. albicans* colonies showed the maximum killing when treated by DC for 12 h (Figure 2C). The SEM showed that the average cell density decreased and cellular structure wrinkled after 12 h-DC treatment (Figure 2D).

For the *C. albicans* biofilms, compared to untreated control, the CFU counting was significantly decreased when treated with 1000 μA DC for 6 h, 12 h and 24 h (Figure 2E), which reached 0.27, 0.94 and 0.97-log killing, respectively. The metabolic activity of DC-stimulated biofilms at 6 h, 12 h and 24 h was significantly lower than the controls(Figure 2F). Crystal violet staining showed significant decrease in the amount of biofilm mass after exposure to DC for 24 h, 48 h and 72 h (Figure 2G). SEM further demonstrated the biofilm density was decreased after continual DC stimulation for 24 h, 48 h and 72 h (Figure 2H). The dead staining (Figure 2I) and agar plates (Figure 2J) of *C. albicans* showed significant killing when treated by DC for 24 h. 

### 3.3. The mRNA and Protein Expression Profiles of DC-Treated Planktonic C. albicans 

**Figure 3:**
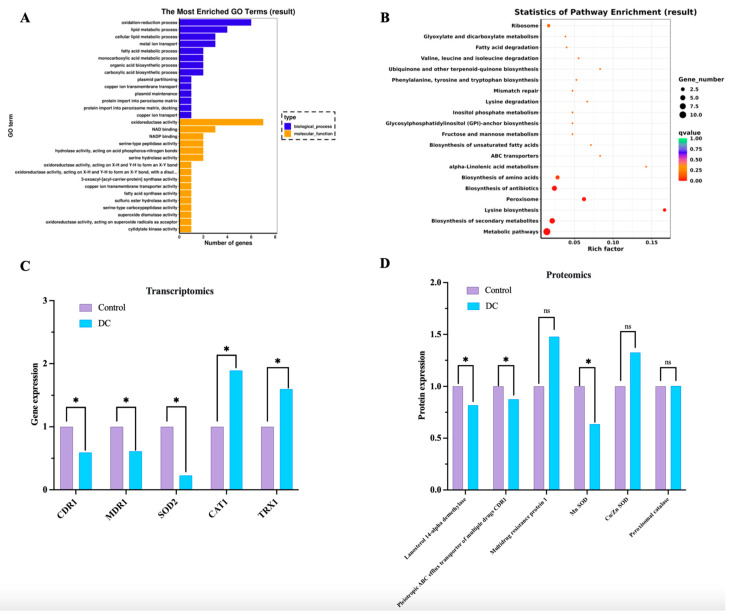
The transcriptomic and proteomic analysis of biological changes on planktonic *C. albicans* after DC treatment. Gene ontology (GO) functional analysis (**A**) and KEGG enrichment (**B**) of differentially expressed genes (DEGs) caused by 1000 μA DC (fold change FDR < 0.05). Selected differentially expressed genes (**C**) and proteins (**D**) involved in drug efflux and oxidative stress were listed. * denotes significant difference of *p* < 0.05. ns denotes no significant difference.

In the planktonic cells of *C. albicans* treated by DC for 6 h, 1200 genes and 49 proteins were found to be differentially expressed. Among these, 509 upregulated genes and 691 downregulated genes were observed, and 18 upregulated proteins and 31 downregulated proteins were involved in the response to DC. After conducting the combined analysis of transcriptomic and proteomic data, the top five GO terms of biological processes were oxidation-reduction process, lipid metabolic process, cellular lipid metabolic process, metal ion transport and fatty acid metabolic process (Figure 3A). Moreover, the top five GO terms of molecular function processes were oxidoreductase activity, NAD binding NADP binding, serine-type peptidase activity, hydrolase activity acting on acid phosphorus-nitrogen bonds and serine hydrolase activity (Figure 3A). The KEGG enrichment analysis revealed that the top 10 enriched pathways included metabolic pathways, biosynthesis of secondary metabolites, lysine biosynthesis, peroxisome, biosynthesis of antibiotics, biosynthesis of amino acids, alpha-linolenic acid metabolism, ABC transporters, biosynthesis of unsaturated fatty acids, and fructose and mannose metabolism (Figure 3B).

As for the pathways of oxidoreductase activity and ABC transporters, the mRNA level of EGR11, CDR1, MDR1 and SOD2 was significantly decreased after DC treatment, and the mRNA level of CAT1 and TRX1 was increased significantly (Figure 3C). The proteomic analysis showed significantly reduced expression of lanosterol 14-alpha demethylase, pleiotropic ABC efflux transporter of multiple drugs CDR1 and Mn SOD, which were in accordance with the mRNA expression changes. The expression of multidrug resistance protein 1, Cu/Zn SOD and peroxisomal catalase increased with no significant difference after DC treatment (Figure 3D).

### 3.4. The Increased ROS Mediated the Killing Effect of DC on Planktonic and Biofilm States of C. albicans 

**Figure 4:**
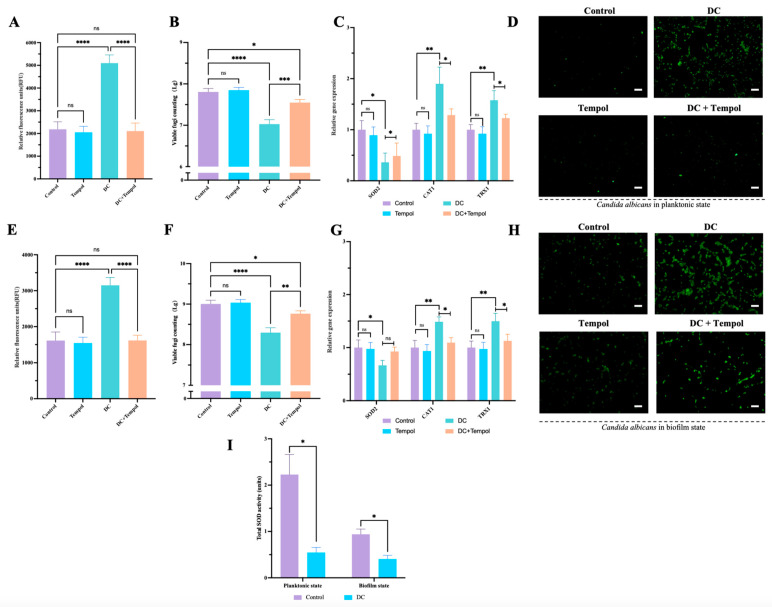
The role of ROS induced by DC on *C. albicans* in planktonic state and biofilm state. The *C. albicans* were treated by 0 μA DC (control), Tempol alone (15 μM for plankton, 30 μM for biofilms), 1000 μA DC combined with or without Tempol (15 μM for the plankton, 30 μM for biofilms), and subjected to analyses of ROS level in the form of RFU ((**A**) in plankton, (**E**) in biofilms), CFU counting ((**B**) in plankton, (**F**) in biofilms), relative gene expression assay ((**C**) in plankton, (**G**) in biofilms) , DCFH-DA staining ((**D**) in plankton, (**H**) in biofilms, scale bar: white line = 50 μm) and total SOD activity comparisons between the control group and DC treatment group (**I**). *, **, ***, **** denote significant difference of *p* < 0.05, *p* < 0.01, *p* < 0.001, *p* < 0.0001, respectively. ns denotes no significant difference.

For the planktonic *C. albicans*, 1000 μA DC treatment for 6 h markedly increased the intracellular ROS content by nearly 2.5-fold (Figure 4A) and caused significant upregulation of CAT1 and TRX1 (Figure 4C). In addition, DC downregulated the mRNA expression of SOD2 (Figure 4C) and exerted ~1-log killing (Figure 4B). Tempol treatment alone did not affect the viable fungi amount and the ROS content. Moreover, compared to DC treatment alone, DC combined with Tempol showed significant downregulation of CAT1 and TRX1 expression, lower oxidative stress and decreased CFU counting (Figure 4A–C). The ROS fluorescence view further confirmed that DC combined with Tempol treatment resulted in lower intracellular oxidative stress than DC only treatment group (Figure 4D).

For the *C. albicans* biofilms, 1000 μA DC treatment for 12 h showed obvious killing effect (less than 1-log killing, Figure 4F). In addition, DC increased the intracellular ROS content by nearly 2-fold (Figure 4E) and led to significant upregulation of CAT1 and TRX1 and downregulation of SOD2 (Figure 4G). Similarly, Tempol did not affect the survival of fungi in biofilms and decreased the ROS content with no greater significance than in the control group (Figure 4E,F). Interestingly, Tempol was able to partially reverse the killing effect of DC on *C. albicans* biofilms and the increased oxidative stress caused by DC treatment. The ROS fluorescence view further demonstrated that DC combined with Tempol treatment showed a lower level of intracellular oxidative stress than the DC only treatment group (Figure 4H). Moreover, DC decreased the total SOD activity of *C. albicans* significantly both in planktonic and biofilm cells (Figure 4I).

### 3.5. DC Showed Synergistic Effect with FLC/AMB against C. albicans in Planktonic and Biofilm States 

**Figure 5:**
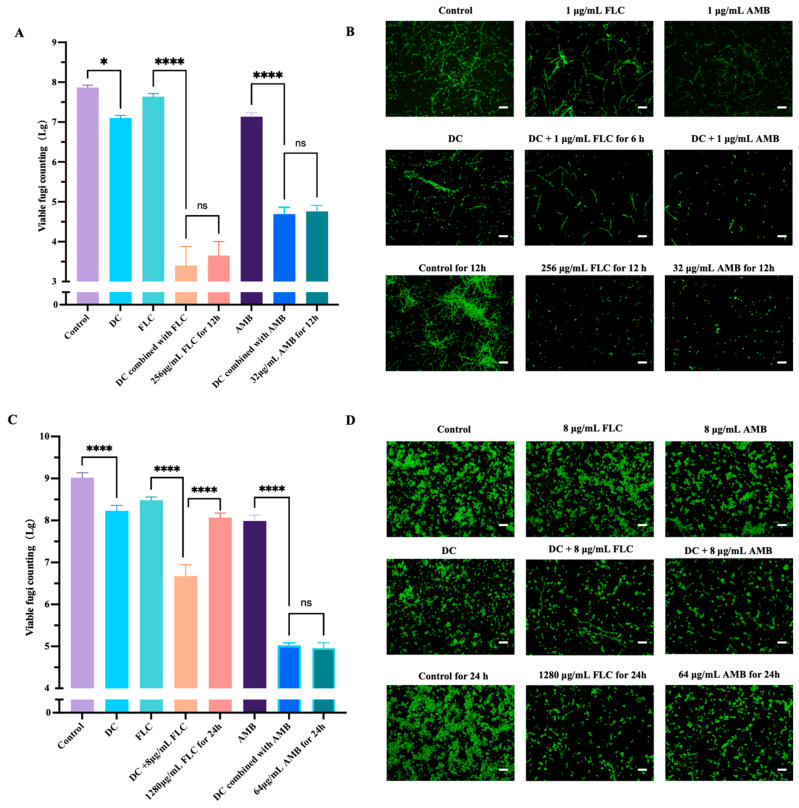
The synergistic effect of DC with FLC/AMB on *C. albicans* in planktonic state and biofilm state. The planktonic *C. albicans* were treated for 6 h by 0 μA DC (control), FLC alone (1 μg/mL), AMB alone (1 μg/mL) and 1000 μA DC combined with or without FLC/AMB (1 μg/mL). Moreover, 0 μA DC for 12 h were set as another negative control group, and 256 μg/mL FLC for 12 h and 32 μg/mL AMB for 12 h were set as the high-dose antifungal group. After stimulation, they were subjected to viable fungi count (**A**) and live staining (**B**). The *C. albicans* biofilms were treated for 12 h by 0 μA DC (control), FLC alone (8 μg/mL), AMB alone (8 μg/mL) and 1000 μA DC combined with or without FLC/AMB (8 μg/mL). Moreover, 0 μA DC for 24 h were set as another negative control group, and 1280 μg/mL FLC for 12 h and 64 μg/mL AMB for 24 h were set as the high-dose antifungal group. After stimulation, they were subjected to viable fungi count (**C**) and live staining (scale bar 50 μm, (**D**)). *, and **** denote significant difference of *p* < 0.05, *p* < 0.0001, respectively. ns denotes no significant difference.

For the planktonic *C. albicans*, 1000 μA DC, 1 μg/mL FLC, 1 μg/mL AMB, DC combined with 1 μg/mL FLC, and DC combined with 1 μg/mL AMB caused significant viable fungi reduction of 0.76, 0.23, 0.73, 4.46 and 3.17 log_10_ CFU/mL compared to control, respectively (Figure 5A). The killing effect of DC combined with 1 μg/mL FLC for 6 h reached a similar level to that achieved by the 256 μg/mL FLC for 12 h treatment. Similarly, the killing effect of DC combined with 1 μg/mL AMB for 6 h reached a similar level to that achieved by the 32 μg/mL AMB for 12 h treatment (Figure 5A). The live staining further confirmed these results (Figure 5B).

For the *C. albicans* biofilms, 1000 μA DC, 8 μg/mL FLC, 8 μg/mL AMB, DC combined with 8 μg/mL FLC, and DC combined with 8 μg/mL AMB caused significant viable fungi reduction of 0.79, 0.54, 1.03, 2.35 and 4.00 log_10_ CFU/mL compared to control, respectively (Figure 5C). The killing effect of DC combined with 8 μg/mL FLC for 12 h was even more effective than 1280 μg/mL FLC for 24 h group. Moreover, the killing effect of DC combined with 8 μg/mL AMB for 12 h reached a similar level to that achieved by the 64 μg/mL AMB for 24 h treatment (Figure 5C). The live staining further confirmed these results (Figure 5D).

### 3.6. DC Increased the Membrane Permeability and Intracellular Drug Concentrations of Planktonic C. albicans 

**Figure 6:**
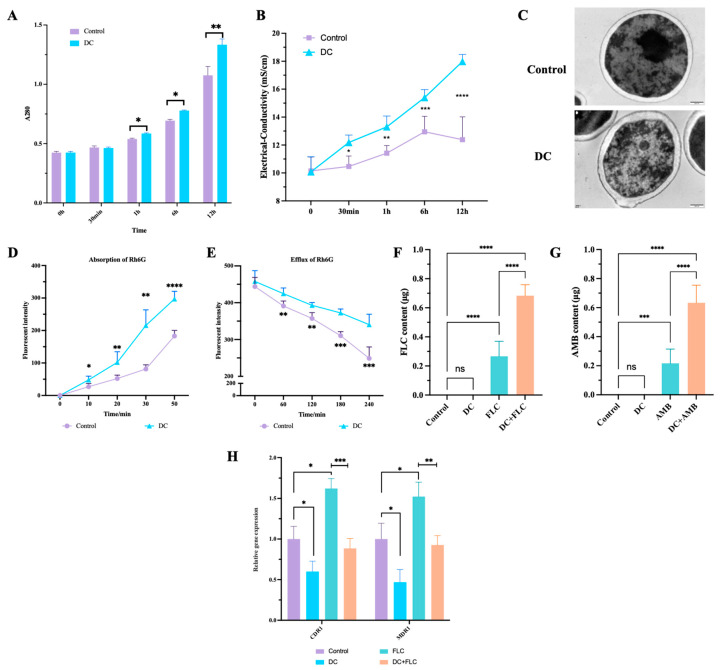
Effect of DC on the absorption and efflux of FLC/AMB. The planktonic *C. albicans* was treated by 0 μA and 1000 μA DC for 0, 0.5, 1, 6 and 12 h. The supernatant after centrifugation was subjected to the A280 assay (**A**) and electrical conductivity assay (**B**). In addition, DC-treated *C. albicans* for 12 h was analyzed by transmission electron microscopy (TEM) (**C**). The absorption (**D**) and efflux (**E**) of Rh6G were detected after DC treatment. The intracellular drug content of FLC (**F**) and AMB (**G**) were detected when treated for 6 h. The relative gene expression of CDR1 and MDR1 were detected after treated by 0 μA alone (control), 1000 μA DC alone, FLC alone, and 1000 μA DC combined with FLC (**H**). *, **, ***, **** denote significant difference of *p* < 0.05, *p* < 0.01, *p* < 0.001, *p* < 0.0001, respectively. ns denotes no significant difference.

The A280 assay showed that the number of external proteins increased with the prolonged stimulation time of DC, with reference to the controls (Figure 6A). The electrical conductivity assay further confirmed DC increased the conductivity of the solution and membrane permeability (Figure 6B); and TEM demonstrated aberrantly shaped membrane and uneven cell walls (Figure 6C). The Rh6G assay showed DC augmented the absorption of Rh6G by *C. albicans* as the DC stimulation time increased from 0 to 50 min, and DC also decreased the efflux of Rh6G continually from 60 to 240 min (Figure 6D,E). The HPLC-DAD/MS analysis showed DC treatment for 6 h significantly increased the cellular drug content of FLC and AMB by 2.56- and 2.92-fold relative to that of the drug alone group, respectively (Figure 6F,G); and DC treatment significantly downregulated the gene expression of CDR1 and MDR1 compared to the control group (Figure 6H). Moreover, DC was able to partially reverse the FLC-induced overexpression of CDR1 and MDR1 (Figure 6H).

### 3.7. SOD2 Gene and Mn SOD Mediated the Killing Effect of DC on AMB-Tolerant Biofilm Persisters of C. albicans 

**Figure 7:**
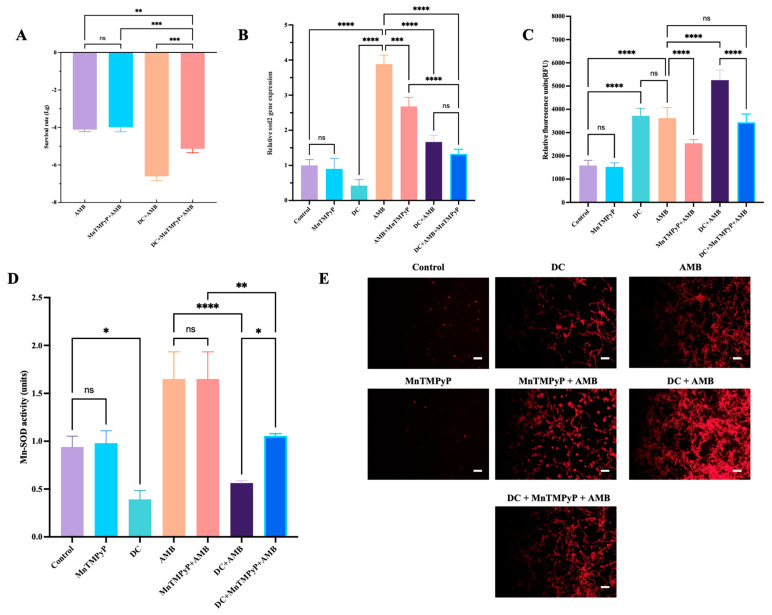
The role of manganese superoxide dismutase (Mn SOD) in the survival rate of AMB-tolerant *C. albicans* biofilm persisters. The *C. albicans* biofilms were treated for 24 h by 256 μg/mL AMB alone (Control), 256 μg/mL AMB combined with 10 μM MnTMPyP, 1000 μA DC or 1000 μA DC with 10 μM MnTMPyP. After the persisters assay, they were subjected to viable fungi count (**A**), relative SOD2 gene expression (**B**), ROS level assay (**C**), Mn SOD activity assay (**D**) and Mito-SOX Red staining (scale bar: white line = 50 μm, (**E**)). *, **, ***, **** denote significant difference of *p* < 0.05, *p* < 0.01, *p* < 0.001, *p* < 0.0001, respectively. ns denotes no significant difference.

Lethal AMB (256 μg/mL) resulted in 0.01% biofilm persisters. After the addition of MnTMPyP, the proportion of persisters increased slightly with no significant difference (Figure 7A). Importantly, the level of persisters decreased 100-fold after adjunctive DC treatment (Figure 7A). However, after the addition of MnTMPyP, the proportion of persisters in the DC combined with MnTMPyP and AMB group increased significantly with reference to the combination of DC and AMB (Figure 7A). Next, we explored the expression of SOD2, Mn SOD activity and ROS level in AMB-tolerant biofilm persisters. After exposure to 256 μg/mL AMB, the oxidative stress significantly increased, accompanied by higher SOD2 gene expression and Mn SOD activity than the control group. DC caused a more significant downregulation of SOD2 gene expression and Mn SOD activity than in the control group, and increased intracellular oxidative stress (Figure 7B–D). Although MnTMPyP alone did not affect SOD2 gene expression significantly, it inhibited the AMB-tolerant higher expression of SOD2 and the increased oxidative stress. Moreover, compared with the DC combined with AMB group, the addition of MnTMPyP weakened the oxidative stress and increased Mn SOD activity. (Figure 7C,D). The Mito-Sox Red staining further confirmed these results, and the treatment with DC or AMB led to mitochondrial ROS production (Figure 7E). MnTMPyP decreased the mitochondrial ROS in *C. albicans* biofilms treated by AMB alone or combined with DC (Figure 7E). 

## 4. Discussion

The present study first showed that constant DC (1000 μA) had a killing effect and synergistic effect with FLC/AMB on planktonic and biofilm states of *C. albicans.* High concentration of FLC and AMB were both able to kill all planktonic cells of *C. albicans*. However, *C. albicans* biofilms were highly resistant to FLC, with less than 1-log CFU reduction when stimulated by 1280 μg/mL FLC. In accordance with our prior research [5], 256 μg/mL AMB killed the majority of *C. albicans* biofilms and about 0.01% biofilm persisters remained alive without inheritable resistance. Notably, when DC and AMB were used in combination, the adjunctive DC treatment was capable of eliminating AMB-tolerant *C. albicans* biofilm persisters. The findings indicate that DC is a novel potential strategy to combat *C. albicans* biofilms and their persisters.

Next, to investigate the mechanism of action of DC, transcriptomic and proteomic analyses were conducted. The results showed that oxidoreductase activity and process in DC-treated *C. albicans* was significantly enriched, suggesting that intracellular oxidative stress induced by DC might play a critical role in killing *C. albicans.* ROS are formed during normal oxygen metabolism and are byproducts of aerobic metabolism, which is necessary for gene expression, cell signaling, and redox homeostasis in certain amounts [28,29]. The antioxidant system is able to scavenge overproduced ROS to maintain adequate amounts of ROS [30]. The balance between the production and scavenging of ROS in cells maintains stability of metabolism and cellular process. The oxidative stress occurs when ROS production overwhelms the scavenging capacity of antioxidants, which can oxidize or damage DNA, proteins, and lipids, thus leading to cellular damage and cell death [21,28,30]. It was reported that DC could induce excessive ROS production and cause bacterial death in *Staphylococcus aureus* and *Staphylococcus epidermidis* biofilms [31,32]. Similar results were observed in this study, in that the cellular ROS level of *C. albicans* was increased after DC treatment and decreased when Tempol was added. Tempol, as a ROS scavenger, is a membrane-permeable and redox-cycling (catalytic) superoxide dismutase mimetic. In this study, Tempol alleviated oxidative stress and was able to decrease ROS levels in DC-treated *C. albicans* to levels comparable to untreated controls. However, the killing efficacy was only partly reversed by the addition of Tempol, suggesting that other mechanisms may also be involved in the antifungal effect of DC. 

To further confirm and validate the role of ROS in DC-associated cell death of *C. albicans*, we examined the gene expression of SOD2, CAT1 and TRX1 by qPCR and Mn SOD activity. Interestingly, we found that DC treatment markedly downregulated SOD2 expression and upregulated CAT1 and TRX1 expression, which could result in decreased total SOD activity and Mn SOD activity. These findings were consistent with the results of the transcriptomic and proteomic analysis. It is well known that Mn SOD is encoded by superoxide dismutase gene 2 (SOD2) and specifically present in the mitochondrial matrix and inner membrane. Mn SOD plays a crucial role in removing superoxide anions and acts as the first line of defense against the mitochondrial oxidative damage [21,33]. Moreover, Tempol was able to reduce oxidative stress and downregulate the expression of SOD2 in DC-treated *C. albicans*. Taken together, the increased oxidation stress induced by DC may be a critical mechanism underlying its antifungal effect.

We found that the combination of DC with FLC/AMB displayed a synergistic effect both in planktonic and biofilm states, the planktonic *C. albicans* being particularly sensitive to this. To understand the mechanisms underlying the synergism, the overall membrane permeability of *C. albicans* was assessed by TEM observation, A280 assay and electrical conductivity. Notably, the cell walls and membranes of *C. albicans* crumpled after DC treatment. Moreover, the protein content and extracellular conductivity rose with increasing treatment time, indicating that membrane permeability increased following DC stimulation. The increased cellular membrane permeability is able to enhance drug uptake and promote drug delivery and treatment efficacy [17,30,34]. Rh6G was used as a tracer of FLC to detect the intracellular FLC concentration, recording the changes in uptake and efflux transport of FLC [27]. As expected, DC treatment could increase the FLC uptake and inhibit the FLC efflux. The HPLC-DAD/MS analysis further confirmed DC led to a nearly 3-fold increase of intracellular FLC relative to the FLC alone group. The overexpression of efflux pumps including CDR1 and MDR1 is one of the most common resistance mechanisms of *C. albicans* to FLC [7,27,35]. The transcriptome and qPCR data revealed that DC significantly suppressed expression of CDR1, which supported the theory of efflux pump inhibition by DC. These findings demonstrated that the synergism between DC and FLC may be related to membrane permeability increase and efflux pump suppression. 

Consistent with our previous study [5], about 0.01% *C. albicans* biofilm persisters remained alive without inheritable resistance after exposure to 256 μg/mL AMB, confirming the limitation of single antifungal treatment in combating *C. albicans* biofilms. In this study, we found that the combination of DC and AMB caused a substantial decrease in the proportion of *C. albicans* biofilm persisters. DC significantly inhibited the expression of SOD2 gene and Mn SOD activity, and resulted in an increased ROS level in *C. albicans* biofilms. Interestingly, the addition of MnTMPyP partly reversed the persister-killing effect of DC. MnTMPyP, as a Mn SOD mimetic, exerted the ROS scavenging effects by increasing the Mn SOD activity in the biofilms. These findings indicate that DC mediated the killing of AMB-tolerant biofilm persisters partly by the inhibition of the SOD2-Mn SOD pathway and the overproduction of ROS. Moreover, our results were consistent with a recent study which showed the significant downregulation of sodB by rifampicin could contribute to greater generation of ROS and lead to clearance of polymyxin B-tolerant persister cells, revealing that the overproduction of ROS may promote the killing of persisters [21]. 

**Figure 8:**
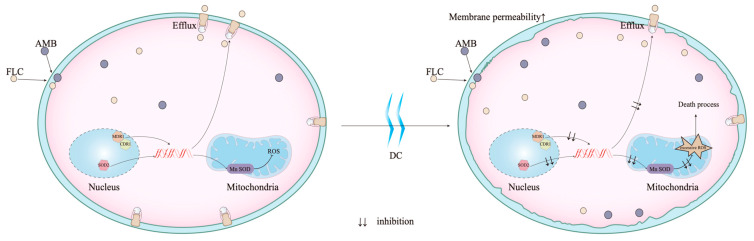
Proposed model for the fungicidal action mechanisms of DC in *C. albicans.* DC increases the membrane permeability, and inhibits the efflux activity by downregulating the gene expressions of CDR1 and MDR1, thus increasing the cellular drug concentrations and killing effect of FLC and AMB. Meanwhile, DC inhibits the expression of SOD2, and decreases Mn SOD activity of scavenging ROS in mitochondria, which leads to an oxidative stress disorder and accelerates the killing of AMB-tolerant persisters.

We found that the increased ROS mediated the killing effect of DC against *C. albicans*. In addition, DC could increase membrane permeability to promote the absorption of FLC and inhibit the efflux pump, thereby showing synergism with FLC against *C. albicans*. DC was also able to downregulate the mRNA expression of SOD2 and inhibit the superoxide scavenging activity of Mn SOD, leading to the overproduction of ROS and thus exerting a killing effect on *C. albicans*-tolerant biofilm persisters. The scheme of the proposed biological mechanisms of DC against *C. albicans* is demonstrated in Figure 8.

Our study had several limitations. To maintain the electric current at a harmless level, the current intensity of DC used in this study was no more than 1000 μA. It is unknown whether larger current intensity could result in a stronger antifungal effect and synergism with FLC/AMB. Moreover, animal studies may be needed prior to its application in the clinical environment in the future. Lastly, owing to the lack of a reliable primary antibody against Mn SOD in *C. albicans* BF-1, the amount of Mn SOD may be difficult to assess quantitatively, further hindering the progress in understanding the nature of DC against *C. albicans*.

In summary, using DC to activate oxidative stress response pathways of SOD2–Mn SOD could harness their potential as efficient molecular targets to control *C. albicans* and persisters in *C. albicans* biofilms. From a certain point of view, DC may be seen as a SOD inhibitor, which induces oxidative stress in *C. albicans* by inhibiting SOD2 gene expression and Mn SOD activity, thus exerting a killing effect on *C. albicans* and AMB-tolerant persisters. The inhibition of the drug efflux pump and increase in membrane permeability caused by DC contribute to the enhanced killing efficacy of FLC and AMB. Hence, our findings suggest that low-intensity DC is a promising approach for controlling *C. albicans* and AMB-tolerant *C. albicans* persisters. In the future, the development of clinically suitable DC devices or charged material may become indicated in effective management of fungal infections. 

## Data Availability

All data supporting the findings of this study are available from the corresponding author upon reasonable request.

## Supplementary Materials

The following supporting information can be downloaded at: https://www.mdpi.com/article/10.3390/antibiotics13060521/s1, Table S1: List of genes and corresponding primers sequence.
