# Supplementary material for: Antifungal Activity, Synergism with Fluconazole or Amphotericin B and Potential Mechanism of Direct Current against Candida albicans Biofilms and Persisters
Source: Antibiotics (Basel). 2024 Jun 3;13(6):521. doi: 10.3390/antibiotics13060521 (PMC11200915; doi:10.3390/antibiotics13060521)
Supplement: Supplementary file 1 [file antibiotics-13-00521-s001.zip › antibiotics-3013401-supplementary.pdf]

Supplementary Table 1. List of genes and corresponding primers sequence.

Gene	Forward sequence of primer (5’-3’)	Reverse sequence of primer (5’-3’)
**SOD2**	ACTACCCGTGCTACTTTGAAC	AATGTATGGTTCAGTAGCGGAG
**CAT1**	ACCCAGAAACTCACTTGAAGG	AGTGTGACCAGAGTAACCATTC
**TRX1**	ATCAATTGGGTTCTTTAGCACAAG	AGCCAAAGCTTGTTTAATAGCAG
**CDR1**	ACAATACAAGACCAGCATCTCC	AGACCCATTACAAGTTGACCG
**MDR1**	AACATTATTATATCGCAAGGCTAAAAGAT	TCCTTCACTTGTGATTCTGTCGTT
**ACT1**	TTGGTGATGAAGCCCAATCC	CATATCGTCCCAGTTGGAAACA
